# College Binge Drinking Associated with Decreased Frontal Activation to Negative Emotional Distractors during Inhibitory Control

**DOI:** 10.3389/fpsyg.2017.01650

**Published:** 2017-09-22

**Authors:** Julia E. Cohen-Gilbert, Lisa D. Nickerson, Jennifer T. Sneider, Emily N. Oot, Anna M. Seraikas, Michael L. Rohan, Marisa M. Silveri

**Affiliations:** ^1^Neurodevelopmental Laboratory on Addictions and Mental Health, McLean Hospital, Belmont MA, United States; ^2^Department of Psychiatry, Harvard Medical School, Boston MA, United States; ^3^Applied Neuroimaging Statistics Laboratory, McLean Hospital, Belmont MA, United States; ^4^Boston University School of Medicine, Boston MA, United States; ^5^McLean Imaging Center, McLean Hospital, Belmont MA, United States

**Keywords:** fMRI, negative emotion, binge drinking, college students, DLPFC, ACC

## Abstract

The transition to college is associated with an increase in heavy episodic alcohol use, or binge drinking, during a time when the prefrontal cortex and prefrontal-limbic circuitry continue to mature. Traits associated with this immaturity, including impulsivity in emotional contexts, may contribute to risky and heavy episodic alcohol consumption. The current study used blood oxygen level dependent (BOLD) multiband functional magnetic resonance imaging (fMRI) to assess brain activation during a task that required participants to ignore background images with positive, negative, or neutral emotional valence while performing an inhibitory control task (Go-NoGo). Subjects were 23 college freshmen (seven male, 18–20 years) who engaged in a range of drinking behavior (past 3 months’ binge episodes range = 0–19, mean = 4.6, total drinks consumed range = 0–104, mean = 32.0). Brain activation on inhibitory trials (NoGo) was contrasted between negative and neutral conditions and between positive and neutral conditions using non-parametric testing (5000 permutations) and cluster-based thresholding (*z* = 2.3), *p* ≤ 0.05 corrected. Results showed that a higher recent incidence of binge drinking was significantly associated with decreased activation of dorsolateral prefrontal cortex (DLPFC), dorsomedial prefrontal cortex (DMPFC), and anterior cingulate cortex (ACC), brain regions strongly implicated in executive functioning, during negative relative to neutral inhibitory trials. No significant associations between binge drinking and brain activation were observed for positive relative to neutral images. While task performance was not significantly associated with binge drinking in this sample, subjects with heavier recent binge drinking showed decreased recruitment of executive control regions under negative versus neutral distractor conditions. These findings suggest that in young adults with heavier recent binge drinking, processing of negative emotional images interferes more with inhibitory control neurocircuitry than in young adults who do not binge drink often. This pattern of altered frontal lobe activation associated with binge drinking may serve as an early marker of risk for future self-regulation deficits that could lead to problematic alcohol use. These findings underscore the importance of understanding the impact of emotion on cognitive control and associated brain functioning in binge drinking behaviors among young adults.

## Introduction

The transition to college is often associated with an escalation in alcohol drinking (Schulenberg and Maggs, 2002), with college students surpassing similarly aged non-students in overall alcohol use and incidence of binge drinking (O’Malley and Johnston, 2002; Carter et al., 2010). Negative consequences associated with binge drinking in college populations include accidental deaths, injuries, assaults, unsafe sex, academic difficulties, and alcohol-related health problems (Hingson et al., 2005, 2006, 2009). Heavy alcohol consumption also is associated with alcohol abuse and dependence (Grant et al., 2009), as is an earlier onset of alcohol use (Hingson et al., 2006, 2009). Binge drinking among college students, coupled with vulnerability of the still-developing brain to the effects of alcohol, may augment the high rate of alcohol use disorders observed within this age group (NSDUH, 2016). Impulsivity, a trait subserved in part by the prefrontal cortex and prefrontal-limbic circuitry, is associated with heavy episodic alcohol consumption and may contribute to the increased rates of binge drinking observed during the transition to college (Nigg et al., 2006; Littlefield et al., 2010; Henges and Marczinski, 2012).

Magnetic resonance imaging (MRI) techniques have demonstrated that brain development continues at a rapid pace through adolescence and into early adulthood, with the majority of alterations occurring in prefrontal cortex (PFC) and in circuits connecting PFC to other brain regions (Sowell et al., 2001; Gogtay et al., 2004; Giorgio et al., 2010). Structural brain changes in PFC have even been detected in as short as a 6-month interval during the first year of college (Bennett and Baird, 2006). PFC development in adolescence and early adulthood is associated with improvements in response inhibition and reduced impulsivity (Johnstone et al., 2005; Durston and Casey, 2006; Jonkman, 2006; Rubia et al., 2007; Stevens et al., 2007). Protracted maturation of PFC and related circuits may therefore render emerging adults, ranging in age from 18 to 24 years, more prone than older individuals to impulsive, risky behaviors, including alcohol consumption and binge drinking (Spear, 2000; Steinberg, 2005; Van Duijvenvoorde et al., 2016). Binge drinking in this age group could in turn, further negatively impact inhibitory control (Sher et al., 1997; Weissenborn and Duka, 2003), given that ongoing plasticity associated with continued development may render PFC and associated functions more vulnerable to the neurotoxic effects of alcohol in younger drinkers (Hermens et al., 2013).

Structural MRI studies have reported increased gray matter volumes in ventral striatum (Howell et al., 2013) and thinner anterior cingulate cortex (ACC; Mashhoon et al., 2014) among college-aged binge drinkers compared to light drinkers, suggesting roles for both reward-processing and regulatory brain regions in binge drinking among youth. Effects of early heavy or binge alcohol use on brain function have been examined longitudinally using fMRI during Go-NoGo tasks, in which inhibitory control is assessed by requiring the suppression of a prepotent response to an infrequent “NoGo” cue in a stream of frequent “Go” cues. In a sample of 16–19-year-olds, lower VMPFC/ACC activation during NoGo trials was found to predict increased substance use and dependence symptoms 18 months later (Mahmood et al., 2013). Similarly, adolescents who later transitioned to heavy drinking were found to show reduced activation in frontal, parietal, subcortical and cerebellar regions during NoGo versus Go trials, relative to adolescents who remained non-drinkers at follow-up (Wetherill et al., 2013). A preliminary prospective study of young adult binge drinkers found correlations between maximum drinks per occasion, activation of a fronto-parietal network during successful inhibition in a Go-NoGo task, and self-reported impulsivity/compulsivity (Worhunsky et al., 2016). While a variety of brain regions have been implicated in altered inhibitory control in alcohol and substance users, a comprehensive review of neuroimaging studies of early alcohol and substance use and abuse reported PFC to be the brain region most frequently impacted by alcohol in youth (Silveri et al., 2016).

Impulsivity, or a lack of inhibitory control, is a broad psychological construct that has multiple sub-components, some of which have been found to be more relevant to drinking frequency, drinking quantity, or negative alcohol-related outcomes in college populations (Cyders et al., 2009). Specifically, among college freshmen who completed the UPPS-P impulsivity scale (Urgency, Premeditation, Perseverance, Sensation Seeking - Positive Urgency; Lynam et al., 2006; Cyders and Smith, 2007, 2008), sensation-seeking was associated with drinking frequency, while positive urgency, or the tendency to act rashly during positive emotions, was related to quantity of drinks consumed per occasion and to negative alcohol-related outcomes (Cyders et al., 2009). Furthermore, negative urgency, or the tendency to act rashly during negative emotions, more than other impulsivity factors, has been found to elevate risk for alcohol abuse in adult populations (Fischer et al., 2004; Verdejo-Garcia et al., 2007; Dick et al., 2011), and also has been linked to poorer Go-NoGo task performance (Dick et al., 2011). Commission errors on the Go-NoGo task have also been linked to binge drinking behaviors among college students (Nigg et al., 2006; Henges and Marczinski, 2012). However, only moderate associations have been found between performance of inhibitory control tasks and self-reported impulsivity (Dick et al., 2011). Use of an emotional Go-NoGo protocol, in which impulsive errors must be avoided in the context of negative and positive emotionally valenced stimuli, may provide a unique, and potentially more sensitive behavioral measure of negative and positive urgency.

The current study used blood oxygen level dependent (BOLD) fMRI to assess brain activation in a sample of 18–19 year-old college freshmen performing an emotional Go-NoGo task, in which participants avoid impulsive errors while ignoring background images selected to elicit negative and positive emotions, as compared to emotionally neutral images. Prior research in healthy young adults has found associations between self-reported impulsivity and activation of dorsomedial PFC (DMPFC) and orbitofrontal cortex (OFC), and between risk taking and activation of OFC and ventromedial PFC (VMPFC) during performance of a Go-NoGo task with neutral or aversive image distractors (Brown et al., 2015). However, specific relationships between inhibitory control in emotional contexts and drinking behaviors in young adults have not yet been studied using fMRI, nor have the neural correlates of the role of positive emotional distraction during inhibitory control been examined.

Whole brain analyses were performed in order to identify brain regions recruited in response to the presence of distracting emotional information during performance of an inhibitory control task. It was hypothesized that emotional relative to neutral distractors would recruit limbic brain areas; specifically, it was anticipated that amygdala would activate in response to negative distractors and ventral striatum would activate in response to positive distractors. Furthermore, inhibitory control efforts during emotional versus neutral background conditions were predicted to require additional executive control and thus elicit additional activation in PFC regulatory regions. A second analysis was conducted to determine whether recruitment of PFC and limbic brain areas during negative versus neutral and positive versus neutral contrasts varied as a function of binge drinking behavior. Given prior evidence of links between heavy drinking and impulsivity in emotional contexts, it was hypothesized that positive associations would be observed between activation of limbic regions during emotional versus neutral inhibitory trials and binge drinking. It also was predicted that a higher incidence of binge drinking would be associated with a failure to recruit executive regions in response to increased inhibitory demands on emotional versus neutral inhibitory trials.

## Materials and Methods

### Participants

Participants included 23 healthy college freshmen (seven male, ages 18–20 years) currently enrolled in a 4-year college program. Alcohol consumption in the prior 3 months was assessed via a Timeline Follow Back interview. Binges were defined as 4+ (female) or 5+ (male) standard drinks in one drinking occasion. Demographic and alcohol consumption measures are summarized in **Table 1**. Subjects were screened and excluded for more than ten lifetime uses of marijuana, more than 25 lifetime uses of tobacco products or any period of regular use (weekly or more frequent use), and any use of illicit drugs other than marijuana. Participants completed urine screening prior to scanning to rule out current psychoactive substance use and pregnancy. Participants were free of neurological disorders, prior head trauma with loss of consciousness, and MRI contraindications such as metal in the body. Participants were required to be alcohol abstinent 48 h prior to scanning. IQ was assessed via the Weschler Abbreviated Scale of Intelligence (WASI, 2 subscale). In a brief family history epidemiology interview, five subjects endorsed a positive family history of alcohol or substance use disorder (father, *n* = 2, or grandparent, *n* = 3).

**Table 1:** Demographic and alcohol use measures.

Measures	Range	Mean	Standard deviation
Age	18.05 – 20.02	18.8	0.4
IQ	93 – 133	115.3	10.3
Number of drinks (past 3 mo)	0 – 103.5	33.8	35.6
Number of binges (past 3 mo)	0 – 19	5.0	6.1
Days since last use^a^	3 – 66	17.7	17.8
Days since last binge^b^	5 – 68	21.0	20.0

*Data represent the range, mean and standard deviation from the total sample (*n* = 23), except: ^a^Estimates reflect data from individuals who reported a minimum of one drink within the past 90 days, *n* = 19; ^b^Estimates reflect data from individuals who reported a minimum of one binge within the past 90 days, *n* = 14*.

The Structured Clinical Interview for DSM-IV (SCID) was used to assess presence or absence of psychiatric disorders. Based on this interview, three participants met criteria for past major depressive disorder (>6 months prior to participation). One participant met criteria for past social phobia (public speaking). One participant met criteria for current social phobia, generalized anxiety disorder, and bulimia nervosa. Two participants met criteria for an alcohol use disorder, which is unsurprising given efforts to recruit heavy drinkers. All participants provided written informed consent prior to participation. This study was approved by the Partners Human Research Committee for McLean Hospital.

### Clinical and Impulsivity Measures

General functioning, including depression and anxiety levels, were assessed via the Counseling Center Assessment of Psychological Symptoms (C-CAPS), a 62-item multi-dimensional mental health assessment tool designed for use in college populations, which includes the following subscales: Depression, Generalized Anxiety, Social Anxiety, Academic Distress, Eating Concerns, Family Distress, Hostility, and Alcohol and Substance Use (C-CAPS, 2011). Problematic alcohol use was assessed via the Alcohol Use Disorder Identification Test (AUDIT), a 10-item screening questionnaire that queries quantity and frequency of alcohol use, binge drinking, dependence symptoms, and alcohol-related problems (Saunders et al., 1993; Babor and Higgins-Biddle, 2000). Self-reported impulsivity was assessed using two survey measures. The Barrett Impulsiveness Scale (BIS-11) provides measures of Attention Impulsivity, Motor Impulsivity and Non-planning Impulsivity (Patton et al., 1995) and the UPPS-P assesses five personality pathways to impulsive behavior: Negative Urgency, (lack of) Premeditation, (lack of) Perseverance, Sensation Seeking, and Positive Urgency (Cyders and Smith, 2007). Survey measures are summarized in **Table 2**.

**Table 2:** Clinical and impulsivity measures.

Measures		Range	Mean	Standard deviation
AUDIT		0 – 17	6.1	5.1
C-CAPS	Depression	0 – 27	10.1	7.3
	Social anxiety	1 – 18	10.9	5.0
	Generalized anxiety	1 – 15	6.0	4.4
	Substance/Alcohol use	0 – 18	6.1	5.0
BIS-11	Attention	12 – 24	16.0	3.1
	Motor	16 – 27	21.6	2.7
	Non-planning	19 – 35	24.1	4.4
	Total	47 – 78	61.7	7.5
UPPS-P	Negative urgency	16 – 45	29.1	7.0
	(Lack of) Premeditation	15 – 34	21.9	5.0
	(Lack of) Perseverance	13 – 31	20.7	4.7
	Sensation seeking	21 – 45	32.3	5.3
	Positive urgency	21 – 46	30.8	7.5

*Data represent range, means and standard deviations from the total sample (*n* = 23)*.

### Go-NoGo Task

During fMRI, subjects performed a task that combines a Go-NoGo task with emotionally arousing background images. This task was adapted from the behavioral task described in Cohen-Gilbert and Thomas (2013) for use with fMRI. As in the prior study, 360 background images were selected from the normatively rated *International Affective Picture System (IAPS)* (Lang et al., 2008) based on valence ratings: 120 highly positive, 120 highly negative, and 120 neutral. Forty images from each of the three valence categories were used to create 120 scrambled images that then served as non-emotional backgrounds that had no discernible image content. Images were presented in blocks of 20 trials of the same background type (positive, negative, neutral or scrambled). Letter stimuli were presented sequentially in a small white box at the center of the background image. Subjects were instructed to respond (button press with thumb) as quickly as possible to every letter except for a target: ‘X’. Xs appeared on 25% of the trials such that participants acquired a prepotent tendency to press and had to actively inhibit responding during NoGo trials. The task was presented via E-prime software synched to the MR scanner via RF pulse. The paradigm used a rapid event-related design, with each trial lasting 1500 ms, including 500 ms of fixation, followed by 350 ms of the background image presented alone, and then 650 ms in which the letter cue and background image were on the screen together. Task jitter was created via distribution of target (NoGo) trials within the stream of Go trials, which were treated as an implicit baseline and not separately modeled (Garavan et al., 2002). Trial order (Go versus NoGo) was optimized for fMRI design efficiency using the Optseq2 program^1^. The task consisted 480 total trials presented in three runs (160 trials/run). This rapid stimulus presentation was used to maintain high levels of inhibitory demand and prevent ceiling effects in this high functioning young adult sample. Task performance measures including accuracy on NoGo trials, accuracy on Go trials, and reaction time on correct Go trials (**Table 3**), were recorded using an MRI-compatible fiber optic response pad (fORP). NoGo trials were randomly distributed throughout each run with the constraint that each 20-trial block contained five NoGo trials.

**Table 3:** Go-NoGo task performance.

Measures	Positive	Negative	Neutral	Scrambled
NoGo accuracy (%)	72.5 (17.6)	72.2 (18.9)	73.6 (15.9)	76.2 (16.3)
Go accuracy (%)	98.4 (2.5)	98.4 (2.2)	98.5 (2.6)	98.7 (2.4)
Go reaction time (ms)	362.9 (32.5)	366.8 (32.2)	358.3 (29.9)	358.9 (28.7)

*Data represent the means and (standard deviations) from the total sample (*n* = 23)*.

### Acquisition and Preprocessing of MRI Data

Data were acquired on a Siemens TIM Trio 3 Tesla scanner (Erlangen, Germany) with a 32-channel head coil. High-resolution structural images were collected using a T1-weighted multiecho Multiplanar Rapidly Acquired Gradient-Echo (ME-MPRAGE) 3D sequence in 4 echoes (TE = 1.64/3.5/5.36/7.22 ms, TR = 2.1 s, TI = 1.1 s, FA = 12°, 176 slices, 1 × 1 × 1.3 mm voxel, acquisition time = 5 min) for registration of functional images to standard space. Whole-brain multiband gradient echo echo-planar imaging (EPI) with BOLD contrast was used to collect fMRI data in three runs (5:13 min/run) (Feinberg et al., 2010). Images were acquired in 54 interleaved, oblique slices (TR/TE/FA = 750 ms/30 ms/52°, FOV = 220, voxel size: 2.8 mm × 2.8 mm × 2.8 mm, multiband = 6, GRAPPA = 2). A fieldmap was acquired at the same resolution and slice locations to allow for offline correction of field inhomogeneities (TR = 1000, TE = 10/12.46 ms, FA = 90°, 2:44 min). Prior to statistical analyses, preprocessing was performed on raw functional images using the FMRIB Software Library (FSL) software v5.0.10 (Smith et al., 2004) including: motion correction, slice-timing correction, non-brain removal, spatial smoothing (FWHM 6mm Gaussian kernel), and grand-mean intensity normalization of the 4D dataset by a single multiplicative factor. Runs began with a 30 s rest block (40 volumes) before task onset, which was removed prior to the current analysis, thus no additional volumes were removed to allow for signal equilibration. ICA AROMA, an independent component analysis-based denoising tool, was then used to remove motion-related components and other components of no interest (e.g., respiration and artifacts) from the fMRI data^2^ (Pruim et al., 2015). No subjects were removed from the analysis due to excessive motion in the scanner. Subject motion was minimal and did not exceed 3 mm (1 voxel) with the exception of a single movement spike slightly above this threshold. Denoised data were then temporally filtered using a Gaussian-weighted least-squares straight line fit with a highpass cutoff = 100 s and underwent fieldmap based distortion correction. fMRI data were registered to MNI152 standard space by first registering the data to the high-resolution structural image using boundary-based registration (BBR) and then transforming into MNI stereotaxic space using the first registration step combined with the registration information from registering the high-resolution structural image to MNI152 standard space, which was done using FNIRT.

### Statistical Analyses

#### Analysis of Task Performance and Survey Data

Performance data were analyzed using repeated-measures analyses of variance (ANOVAs) and paired samples *t*-tests. Correlations were conducted to test for relationships between drinking measures, survey measures, and task performance using Pearson’s correlations. These statistical analyses were carried out using SPSS (version 23.0).

#### Analysis of fMRI Data

FEAT v6.00 was used to conduct hierarchical voxel-wise general linear model (GLM) analyses. First-level modeling was conducted for each of the three runs for each participant. NoGo trials within the four background conditions (positive, negative, neutral, scrambled) were each modeled as separate regressors, convolved with a gamma function. Temporal derivatives were also included in the model. Go trials were treated as an implicit baseline of tonic, task-related activity and were not modeled separately. Contrasts of parameter estimates (COPE) were calculated between positive and neutral conditions and between negative and neutral conditions. The scrambled condition was not examined in the current analysis due to a lack of specific hypotheses regarding brain activation for this condition in relation to alcohol use. For each COPE, the three task runs were combined for each participant using a second-level fixed effects GLM to create averaged COPE maps.

In order to identify brain regions recruited in response to increased emotional distraction during inhibitory control, a third-level whole brain voxel-wise single-group GLM was conducted across all participants for each of the (second-level) contrasts of interest. Estimation and inference were done using FSL Randomise, with non-parametric permutation testing (5000 permutations) and cluster-based thresholding (*z* = 2.3). Results of the whole-brain analysis are shown for a significance level of *p* ≤ 0.05, corrected for family wise error. Use of non-parametric permutation testing obviates any concerns related to inflated false-positive rates in fMRI inference for spatial extent that were described in a recent report (Eklund et al., 2016).

In order to test our hypotheses related to associations of prefrontal and limbic brain regions with recent binge drinking, a second group-level GLM was conducted for each contrast of interest in which the number of binges in the past 3 months was included as a predictor. A mask encompassing the brain regions hypothesized to be most impacted by binge drinking – PFC, amygdala, and nucleus accumbens – was applied for this analysis. The mask was constructed by combining the frontal cortex region, defined by MNI Structural Atlas, with the bilateral amygdala and bilateral nucleus accumbens regions from the Harvard-Oxford Subcortical Structure Atlas. Because binge drinking varied between males and females within this sample and because biological sex may influence the neurological impact of alcohol use, sex was included as a covariate of non-interest. Estimation and inference were performed using FSL Randomise (5000 permutations) with cluster-based thresholding (*z* = 2.3). Results are shown for a significance level of *p* ≤ 0.05, corrected for family wise error.

## Results

### Behavioral, Impulsivity and Clinical Results

Repeated-measures ANOVAs were used to examine the impact of background distractor images (positive, negative, neutral or scrambled) on task performance measures: accuracy on NoGo trials, accuracy on Go trials, and reaction time on correct Go trials. These analyses showed no significant effects of background on accuracy on either Go or NoGo trials. A significant main effect of background on reaction time was observed, *F*(3,66) = 3.98, *p* = 0.011. *Post hoc* paired samples *t*-tests revealed significantly longer reaction times on negative trials relative to neutral trials, *t*(22) = 3.45, *p* = 0.002, and scrambled trials, *t*(22) = 2.85, *p* = 0.009. Task performance measures were not significantly correlated with past 3 months’ binges or number of drinks or with drinking outcome measures (AUDIT and CCAPS substance and alcohol use subscale). Likewise, task performance was not significantly associated with self-reported impulsivity (BIS-11 and UPPS-P) and no significant correlations were found between drinking measures and self-reported impulsivity. As would be expected, AUDIT scores were significantly positively related to past 3 months’ binges, *r* = 0.701, *p* < 0.001, and past 3 months’ drinks, *r* = 0.757, *p* < 0.001. Similarly, the alcohol and substance use problems score on the C-CAPS was significantly related to past 3 months’ binges, *r* = 0.643, *p* = 0.001, and drinks, *r* = 0.772, *p* < 0.001.

### fMRI Results

#### Emotion Related Activation during Inhibitory Control

In order to identify brain regions recruited in response to the addition of task-irrelevant emotional information during conditions demanding inhibitory control (NoGo trials), contrasts of negative versus neutral and positive versus neutral conditions were first examined in the full sample.

##### Negative versus neutral background conditions

**FIGURE 1:**
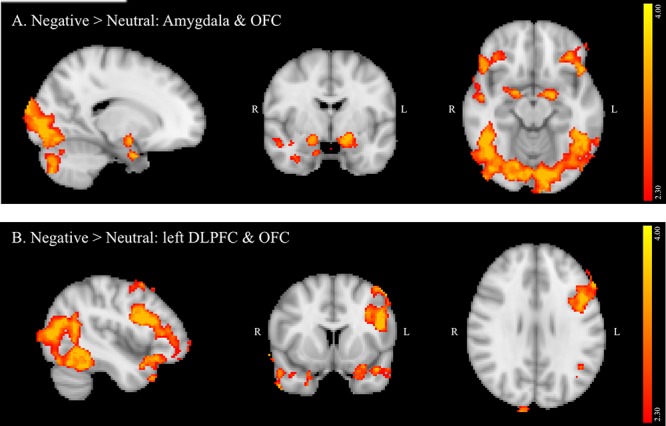
**(A,B)** Region showing greater activation to negative versus neutral background conditions during NoGo trials. MNI co-ordinates: **(A)** *x* = 17, *y* = –4, *z* = –15; **(B)** *x* = –45, *y* = 10, *z* = 28.

A contrast of negative and neutral NoGo trials revealed a single spatially extended cluster comprised of multiple brain regions that showed greater activation during negative versus neutral background conditions. Regions within the cluster included bilateral OFC (**Figures 1A,B**) and amygdala (**Figure 1A**), as well as a large left-lateralized prefrontal area comprising left Inferior Frontal Gyrus (IFG), Middle Frontal Gyrus (MFG), precentral gyrus, and frontal pole (**Figure 1B**). In addition to PFC and limbic regions, activation was observed bilaterally in the temporal pole and inferior temporal gyrus, and in the right superior temporal gyrus. Activation also was observed in many visual processing regions, including temporal and occipital fusiform cortex, lateral occipital cortex and occipital pole, and in the cerebellum. A summary of the anatomical locations of local maxima for this cluster is provided in **Table 4**. No significant regions of activation were revealed in the neutral > negative contrast.

**Table 4:** Local maxima of activation for all participants: negative > neutral contrast.

Region	Side	Volume (mm^3^)	z-max df = 22	MNI Coordinates		
				*x*	*y*	*z*
Extended region including:		49166				
Frontal pole	L		3.54	-54	44	-12
Frontal orbital cortex/Frontal pole	R		3.54	38	34	-22
Inferior frontal gyrus	L		3.54	-46	34	12
Temporal pole	R		3.54	44	26	-34
Temporal pole/Frontal orbital cortex	L		3.54	-28	18	-30
Temporal pole	R		3.54	54	10	-40
Parahippocampal gyrus/Amygdala	R		3.54	20	0	-30
Amygdala	R		3.54	20	-2	16
Temporal fusiform cortex	R		3.54	36	-6	-32
Cerebellum	R		3.54	36	-76	-52

##### Positive versus neutral background conditions

**FIGURE 2:**
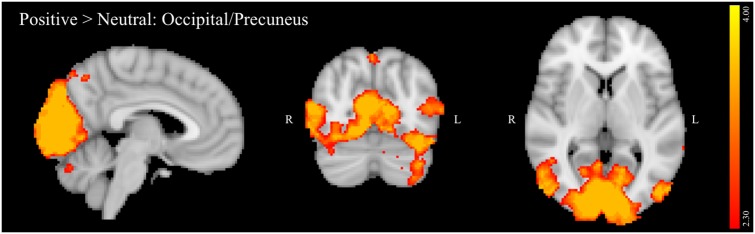
Region showing greater activation to positive versus neutral background conditions during NoGo trials. MNI co-ordinates: *x* = 5, *y* = –71, *z* = 5.

The positive versus neutral contrast did not reveal any PFC regions that were significantly activated in positive relative to neutral distractor conditions. Furthermore, contrary to one of the study hypotheses, no activation was observed in the ventral striatum on this contrast. Activated regions (**Figure 2**) included: inferior temporal gyrus, temporal and occipital fusiform cortex, lateral occipital cortex, occipital pole, cuneus, precuneus, and cerebellum. A summary of the anatomical locations of local maxima for this cluster is provided in **Table 5**. No significant regions of activation were revealed in the neutral > positive contrast.

**Table 5:** Local maxima for all participants: positive > neutral contrast.

Region	Side	Volume (mm^3^)	z-max df = 22	MNI coordinates		
				*x*	*y*	*z*
Extended region including:		43086				
Inferior temporal gyrus/Temporal Occipital fusiform cortex/Cerebellum	R		3.54	48	-46	-30
Cerebellum	L		3.54	-38	-70	-40
Precuneous cortex	B		3.54	2	-74	54
Cerebellum	R		3.24	16	-78	-40
Lateral occipital cortex	L		3.54	-48	-86	2

#### Binge Drinking Related Activation

**FIGURE 3:**
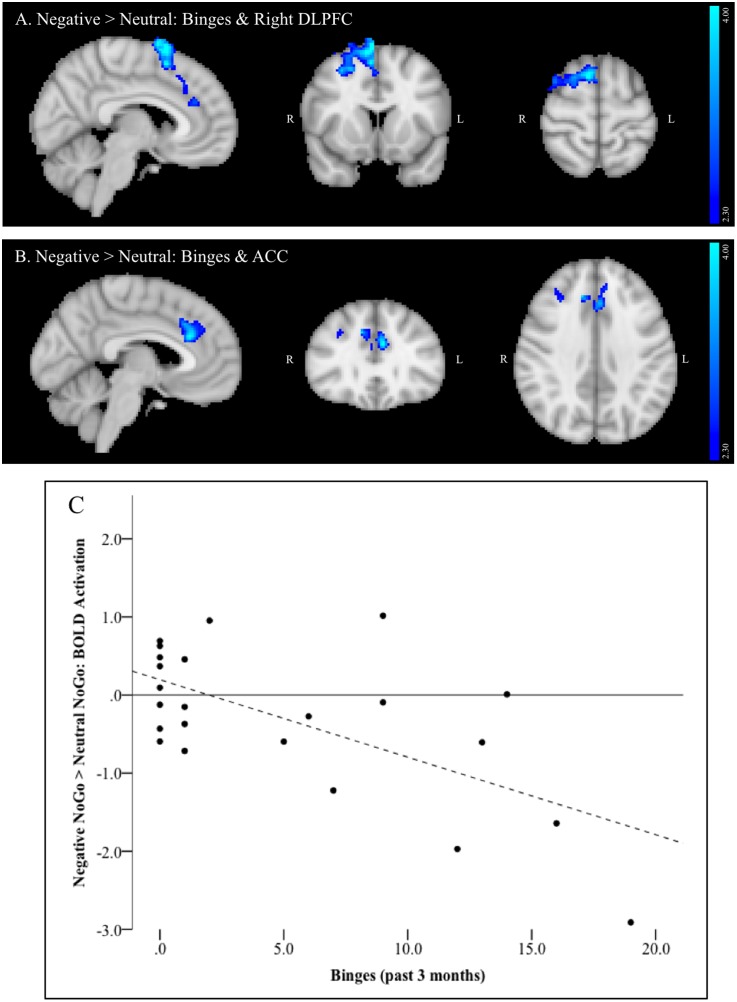
**(A–C)** Region negatively associated with number of binges (past 3 months) in the negative NoGo versus neutral NoGo contrast. MNI co-ordinates: **(A)** *x* = 6, *y* = 12, *z* = 63; **(B)** *x* = –5, *y* = 30, *z* = 36; **(C)** Scatterplot of activation within this region (negative NoGo versus neutral NoGo contrast) versus number of binges (past 3 months).

For the negative > neutral contrast, a single large cluster of activation comprised of multiple brain regions was significantly negatively correlated with past 3 months’ binges. Areas within the cluster included right dorsolateral PFC (DLPFC) and dorsomedial PFC (DMPFC, **Figure 3A**), including MFG and superior frontal gyrus (SFG) and a relatively small region of the juxtapositional lobule cortex (formerly pre-motor cortex). The cluster also included bilateral ACC (**Figure 3B**) and bilateral paracingulate cortex. **Figure 3C** depicts the negative linear relationship between strength of activation within this cluster and number of past 3 months’ binges.

## Discussion

Drinking potentiates impulsive actions, particularly during emotion-laden circumstances, which may in turn lead to continued drinking to binge levels. Such behaviors have significant and lasting deleterious effects on brain regions critical to cognitive and emotional regulation, particularly in youth. The current study sample consisted of college freshmen who represented a continuum of drinking that ranged from not yet initiated through meeting criteria for alcohol use disorder. In this group, activation of DLPFC, DMPFC and ACC during a response inhibition task with negative emotion-based distractors was found to decrease with greater recent heavy episodic alcohol use. This reduced activation may reflect a failure to bring regulatory brain regions online when negative emotional distractors elevate cognitive control demands. In other words, with increased binge drinking, task-irrelevant negatively valenced information increasingly damps down recruitment of brain regions implicated in executive control and error monitoring (Carter et al., 1998; Smith and Jonides, 1999; Van Veen and Carter, 2002; Ridderinkhof et al., 2004). Binge-related activation effects were specific only to the negatively valenced distractor condition, as no relationship to binge consumption was evident for activation during exposure to positively valenced distractors.

In the full sample, though not specifically related to binge drinking, negative relative to neutral distractor conditions also significantly recruited amygdala, OFC, and left DLPFC (IFG and MFG) during NoGo trials. Given that the amygdala has been reliably associated with threat monitoring and processing of negative emotion (LeDoux, 2003), activation in this region suggests that negatively valenced background images effectively elicited negative emotion in the context of concurrent inhibitory control despite instructions to ignore image content. Increased activation of DLPFC and OFC on negative versus neutral NoGo trials supports the hypothesis that impulse control becomes more challenging in the context of these negative distractors, requiring increased recruitment of regions implicated in successful response inhibition (Spinella, 2004). Furthermore, lateralization of the observed DLPFC activation aligns with studies suggesting that left PFC is particularly crucial in top-down regulation of negative emotion (Bruder et al., 2017). Current findings also are congruent with a meta-analysis of tasks probing the interaction of emotion and cognitive control, which found that task irrelevant emotion consistently recruited clusters in SFG, MFG and IFG, and amygdala, though this analysis did not differentiate between valence or modality of emotional distractors (Cromheeke and Mueller, 2014). Recruitment of visual processing areas by the current task, including occipital pole, lateral occipital, and fusiform cortices in both negative versus neutral and positive versus neutral contrasts in the current study likely reflects increased visual processing of the emotional images due to their higher salience relative to neutral background images. In contrast to negative distractors, positive distractors failed to elicit significant recruitment of predicted limbic and PFC regions. The absence of ventral striatum activation may reflect that positively valenced images in the current study were not rewarding *per se*, since this region is most reliably implicated in the processing of reward (Knutson et al., 2001). Examining the impact of these stimuli during the context of a challenging and potentially frustrating task – and during inhibitory trials in particular – may have further reduced activation of ventral striatum. Absence of PFC activation further suggests that positive images may have been less effective distractors, given the lack of an impact on inhibitory control demands. This may, in turn, inform why binge drinking did not predict activation on the positive versus neutral contrast.

With regard to overall task performance, as in Cohen-Gilbert and Thomas (2013), neither positive nor negative emotional images impacted accuracy on Go or NoGo trials in this task, however, slower response times were observed on negative relative to neutral trials. This slowed responding suggests that negative images were more salient than non-emotional distractors, pulling attention away from the assigned inhibitory control task. Recruitment of PFC regions in response to negative versus neutral, but not positive versus neutral distractors, further supports the possibility that negative image distractors in this task pose a greater challenge to cognitive control. Finally, brain activation differences associated with binge drinking on the negative versus neutral contrast were observed in the absence of effects on task performance, as drinking measures were not significantly associated with accuracy or reaction time measures. The reduced impact of positive distractors on performance may also contribute to the absence of the hypothesized impact of binge drinking on frontal and limbic brain activation during positive NoGo trials.

Elevated impulsivity has been tied to increased alcohol use and heavy episodic alcohol consumption among college students (Cyders et al., 2009) and the tendency toward rash action is further increased by alcohol consumption. However, despite prior research suggesting relationships between impulsivity and binge drinking (Nigg et al., 2006; Henges and Marczinski, 2012), no significant relationships between self-report or behavioral measures of impulsivity and drinking behavior were observed in the current study. Several factors may contribute to this. First, response inhibition has been found to serve as an incremental predictor of alcohol and substance use, but accounts for only a small amount of variance in outcomes (Nigg et al., 2006). Studies reporting relationships between survey or strictly behavioral measures of impulsivity and drinking behavior typically feature considerably larger sample sizes and thus have the power to detect more modest effects. Furthermore, a meta-analysis of commission errors on Go-NoGo tasks in substance users found evidence of deficits in alcohol dependent individuals, but not in heavy drinkers who did not meet criteria for dependence, suggesting the impact of alcohol on inhibitory control may be dose-dependent (Smith et al., 2014). There also is evidence that relationships between alcohol abuse and impulsivity are at least partly driven by common comorbid psychopathological symptoms (Whiteside and Lynam, 2003).

In previous work, relative to age-matched light drinkers, healthy college aged binge drinkers demonstrated no significant differences on clinical measures of depression, anxiety, impulsivity or emotional intelligence, or across multiple cognitive domains, with the exception of modestly lower verbal learning scores in binge drinkers (Sneider et al., 2013). In contrast, significant binge-related structural and neurochemical differences were observed, with the binge group exhibiting a thinner cortex (Mashhoon et al., 2014) and lower brain GABA metabolite levels (Silveri et al., 2014), both of which were specific to the frontal lobe. These multimodal results suggest that while the frontal cortex is differentially sensitive to binge versus light alcohol consumption, observed neurobiological alterations associated with binge drinking may not necessarily manifest as clinical symptoms or cognitive impairments. Results of the current study extend the current literature to include functional differences on an executive functioning task requiring PFC activation in the presence of negatively valenced stimuli, which was negatively linearly associated with increasing numbers of binges. Among a number of possible interpretations, altered neuroimaging measures may reflect acute neurotoxic effects of binge drinking, which could increase risk for future adverse outcomes (or resolve with age-related declines in problematic use, i.e., “maturing out”). Alternatively, given a clinically and cognitively healthy status, a neurobiological signature of binge drinking could reflect protective adaptations to chronic, intermittent alcohol exposure. A third possibility is a combination of these interpretations: that neurobiological adaptations protect the young brain from immediate functional impairment, while simultaneously increasing risk for future adverse outcomes. Similarly in this sample, the college freshmen participants were healthy, high-functioning individuals, many of whom did not begin heavy drinking until the transition to college. Thus, similar task performance despite the presence of brain activation differences may reflect successful compensatory mechanisms or brain differences in this type of cohort that may still be too subtle to manifest as a significant behavioral difference.

There are some minor limitations, besides a modest sample size, that should be considered when interpreting results. The task design, while minimally altering the behavioral task as presented outside the scanner, does not allow for separate modeling of Go trial activation, which prevents the direct comparison of inhibitory to non-inhibitory trials within each background condition. However, this trade-off allowed us to maintain high levels of inhibitory demand and prevent ceiling effects in this high functioning young adult sample, and equally important, allowed us to test our main hypotheses related to the impact of emotionally valenced stimuli on inhibitory processing. Drinking patterns varied between males and females in the study sample, with included males tending to be the light drinkers, due in part to heavier drinking males being excluded due to co-marijuana use. With a low number of males in the overall sample, the influence of sex differences could not be investigated. Sex likely plays a significant role in specifying relationships between emotion, response inhibition, binge drinking and neurobiology (Townshend and Duka, 2005; Nederkoorn et al., 2009) and will be important to study in future work.

## Conclusion

The current study provides evidence that recent binge drinking is associated with decreased activation of key executive regions in the presence of negative, but not positive, emotional distractors during performance of an inhibitory control task. This reduced activation may indicate a failure to engage cognitive control regions to regulate emotion processing and may serve as an early marker of risk for future self-regulation deficits associated with problematic alcohol use. These findings underscore the importance of understanding the impact of emotion on cognitive control and associated brain functioning in binge drinking behaviors among emerging adults. Brain activation patterns in this sample of college freshmen are being examined as potential predictors of subsequent alcohol consumption patterns throughout college, as follow-up assessments are being conducted yearly after the baseline imaging assessment. Longitudinal data will help elucidate whether activation differences are direct consequences of recent alcohol use or of a combination of related environmental and neurobiological factors. These data will also inform whether this neurobiological signature is predictive of longer-term problematic use.

## Ethics Statement

The procedures reported in this study were approved by the Research Ethics Boards relevant to McLean Hospital, and were carried out in accordance with the Declaration of Helsinki.

## Author Contributions

JC-G and MS conceptualized the study. EO and AS contributed to study recruitment, coordination and data collection. MR implemented the fMRI scanning sequence. JC-G, LN, JS, EO, and AS conducted data processing and analyses. JC-G, LN, and MS drafted the manuscript. All co-authors made contributions, edited and approved the final manuscript.

## Conflict of Interest Statement

The authors declare that the research was conducted in the absence of any commercial or financial relationships that could be construed as a potential conflict of interest.

## Footnotes

1 http://surfer.nmr.mgh.harvard.edu/optseq/

2 https://github.com/rhr-pruim/ICA-AROMA

## References

[B1] Babor T. F. Higgins-Biddle J. C. (2000). Alcohol screening and brief intervention: dissemination strategies for medical practice and public health. *Addiction* 95 677–686. 10.1046/j.1360-0443.2000.9556773.x 10885042

[B2] Bennett C. M. Baird A. A. (2006). Anatomical changes in the emerging adult brain: a voxel-based morphometry study. *Hum. Brain Mapp.* 27 766–777. 10.1002/hbm.20218 16317714 PMC6871409

[B3] Brown M. R. Benoit J. R. Juhás M. Lebel R. Mackay M. Dametto E. (2015). Neural correlates of high-risk behavior tendencies and impulsivity in an emotional Go/NoGo fMRI task. *Front. Syst. Neurosci.* 9:24 10.3389/fnsys.2015.00024 PMC4354310 25805975

[B4] Bruder G. E. Stewart J. W. Mcgrath P. J. (2017). Right brain, left brain in depressive disorders: clinical and theoretical implications of behavioral, electrophysiological and neuroimaging findings. *Neurosci. Biobehav. Rev.* 78 178–191. 10.1016/j.neubiorev.2017.04.021 28445740

[B5] Carter A. C. Brandon K. O. Goldman M. S. (2010). The college and noncollege experience: a review of the factors that influence drinking behavior in young adulthood. *J. Stud. Alcohol Drugs* 71 742–750. 10.15288/jsad.2010.71.742 20731981 PMC2930506

[B6] Carter C. S. Braver T. S. Barch D. M. Botvinick M. M. Noll D. Cohen J. D. (1998). Anterior cingulate cortex, error detection, and the online monitoring of performance. *Science* 280 747–749. 10.1126/science.280.5364.747 9563953

[B7] C-CAPS (2011). *Center for Collegiate Mental Health, Annual Report*. University Park, PA: Student Health Center.

[B8] Cohen-Gilbert J. E. Thomas K. M. (2013). Inhibitory control during emotional distraction across adolescence and early adulthood. *Child Dev.* 84 1954–1966. 10.1111/cdev.12085 23506340 PMC3688699

[B9] Cromheeke S. Mueller S. C. (2014). Probing emotional influences on cognitive control: an ALE meta-analysis of cognition emotion interactions. *Brain Struct. Funct.* 219 995–1008. 10.1007/s00429-013-0549-z 23563751

[B10] Cyders M. A. Flory K. Rainer S. Smith G. T. (2009). The role of personality dispositions to risky behavior in predicting first-year college drinking. *Addiction* 104 193–202. 10.1111/j.1360-0443.2008.02434.x 19149813 PMC2653206

[B11] Cyders M. A. Smith G. T. (2007). Mood-based rash action and its components: positive and negative urgency. *Pers. Individ. Dif.* 43 839–850. 10.1016/j.paid.2007.02.008

[B12] Cyders M. A. Smith G. T. (2008). Emotion-based dispositions to rash action: positive and negative urgency. *Psychol. Bull.* 134 807–828. 10.1037/a0013341 18954158 PMC2705930

[B13] Dick D. M. Smith G. Olausson P. Mitchell S. H. Leeman R. F. O’Malley S. S. (2011). Understanding the construct of impulsivity and its relationship to alcohol use disorders. *Addict. Biol.* 15 217–226. 10.1111/j.1369-1600.2009.00190.x PMC2895996 20148781

[B14] Durston S. Casey B. J. (2006). What have we learned about cognitive development from neuroimaging? *Neuropsychologia* 44 2149–2157. 16303150 10.1016/j.neuropsychologia.2005.10.010

[B15] Eklund A. Nichols T. E. Knutsson H. (2016). Cluster failure: why fMRI inferences for spatial extent have inflated false-positive rates. *Proc. Natl. Acad. Sci. U.S.A.* 113 7900–7905. 10.1073/pnas.1602413113 27357684 PMC4948312

[B16] Feinberg D. A. Moeller S. Smith S. M. Auerbach E. Ramanna S. Glasser M. F. (2010). Multiplexed echo planar imaging for sub-second whole brain FMRI and fast diffusion imaging. *PLOS ONE* 5:e15710 10.1371/journal.pone.0015710 PMC3004955 21187930

[B17] Fischer S. Anderson K. G. Smith G. T. (2004). Coping with distress by eating or drinking: role of trait urgency and expectancies. *Psychol. Addict. Behav.* 18 269–274. 10.1037/0893-164X.18.3.269 15482082

[B18] Garavan H. Ross T. Murphy K. Roche R. Stein E. (2002). Dissociable executive functions in the dynamic control of behavior: inhibition, error detection, and correction. *Neuroimage* 17 1820–1829. 10.1006/nimg.2002.1326 12498755

[B19] Giorgio A. Watkins K. E. Chadwick M. James S. Winmill L. Douaud G. (2010). Longitudinal changes in grey and white matter during adolescence. *Neuroimage* 49 94–103. 10.1016/j.neuroimage.2009.08.003 19679191

[B20] Gogtay N. Giedd J. N. Lusk L. Hayashi K. M. Greenstein D. Vaituzis A. C. (2004). Dynamic mapping of human cortical development during childhood through early adulthood. *Proc. Natl. Acad. Sci. U.S.A.* 101 8174–8179. 10.1073/pnas.0402680101 15148381 PMC419576

[B21] Grant J. D. Agrawal A. Bucholz K. K. Madden P. A. Pergadia M. L. Nelson E. C. (2009). Alcohol consumption indices of genetic risk for alcohol dependence. *Biol. Psychiatry* 66 795–800. 10.1016/j.biopsych.2009.05.018 19576574 PMC3077105

[B22] Henges A. L. Marczinski C. A. (2012). Impulsivity and alcohol consumption in young social drinkers. *Addict. Behav.* 37 217–220. 10.1016/j.addbeh.2011.09.013 21981824 PMC3230724

[B23] Hermens D. F. Lagopoulos J. Tobias-Webb J. De Regt T. Dore G. Juckes L. (2013). Pathways to alcohol-induced brain impairment in young people: a review. *Cortex* 49 3–17. 10.1016/j.cortex.2012.05.021 22789780

[B24] Hingson R. W. Heeren T. Winter M. Wechsler H. (2005). Magnitude of alcohol-related mortality and morbidity among U.S. college students ages 18-24: changes from 1998 to 2001. *Annu. Rev. Public Health* 26 259–279. 10.1146/annurev.publhealth.26.021304.144652 15760289

[B25] Hingson R. W. Heeren T. Winter M. R. (2006). Age at drinking onset and alcohol dependence: age at onset, duration, and severity. *Arch. Pediatr. Adolesc. Med.* 160 739–746. 10.1001/archpedi.160.7.739 16818840

[B26] Hingson R. W. Zha W. Weitzman E. R. (2009). Magnitude of and trends in alcohol-related mortality and morbidity among U.S. college students ages 18-24, 1998-2005. *J. Stud. Alcohol Drugs Suppl.* S16 12–20. 10.15288/jsads.2009.s16.12 PMC2701090 19538908

[B27] Howell N. A. Worbe Y. Lange I. Tait R. Irvine M. Banca P. (2013). Increased ventral striatal volume in college-aged binge drinkers. *PLOS ONE* 8:e74164 10.1371/journal.pone.0074164 PMC3785474 24086317

[B28] Johnstone S. J. Pleffer C. B. Barry R. J. Clarke A. R. Smith J. L. (2005). Development of inhibitory processing during the Go/NoGo task: a behavioral and event-related potential study of children and adults. *J. Psychophysiol.* 19 11–23. 10.1027/0269-8803.19.1.11

[B29] Jonkman L. M. (2006). The development of preparation, conflict monitoring and inhibition from early childhood to young adulthood: a Go/Nogo ERP study. *Brain Res.* 1097 181–193. 10.1016/j.brainres.2006.04.064 16729977

[B30] Knutson B. Adams C. M. Fong G. W. Hommer D. (2001). Anticipation of increasing monetary reward selectively recruits nucleus accumbens. *J. Neurosci.* 21 RC159. 10.1523/JNEUROSCI.21-16-j0002.2001 PMC6763187 11459880

[B31] Lang P. J. Bradley M. M. Cuthbert B. N. (2008). *International Affective Picture System (IAPS): Affective Ratings of Pictures and Instruction Manual*. Technical Report A-8 Gainesville, FL: University of Florida.

[B32] LeDoux J. (2003). The emotional brain, fear, and the amygdala. *Cell Mol. Neurobiol.* 23 727–738. 10.1023/A:1025048802629 14514027 PMC11530156

[B33] Littlefield A. K. Sher K. J. Steinley D. (2010). Developmental trajectories of impulsivity and their association with alcohol use and related outcomes during emerging and young adulthood I. *Alcoholism* 34 1409–1416. 10.1111/j.1530-0277.2010.01224.x 20528822 PMC4260532

[B34] Lynam D. R. Smith G. T. Whiteside S. P. Cyders M. A. (2006). *The Upps-P: Assessing Five Personality Pathways to Impulsive Behavior*. West Lafayette, IN: Purdue University.

[B35] Mahmood O. M. Goldenberg D. Thayer R. Migliorini R. Simmons A. N. Tapert S. F. (2013). Adolescents’ fMRI activation to a response inhibition task predicts future substance use. *Addict. Behav.* 38 1435–1441. 10.1016/j.addbeh.2012.07.012 23006248 PMC3493685

[B36] Mashhoon Y. Czerkawski C. Crowley D. J. Cohen-Gilbert J. E. Sneider J. T. Silveri M. M. (2014). Binge alcohol consumption in emerging adults: anterior cingulate cortical “thinness” is associated with alcohol use patterns. *Alcohol. Clin. Exp. Res.* 38 1955–1964. 10.1111/acer.12475 24961871 PMC4107054

[B37] Nederkoorn C. Baltus M. Guerrieri R. Wiers R. W. (2009). Heavy drinking is associated with deficient response inhibition in women but not in men. *Pharmacol. Biochem. Behav.* 93 331–336. 10.1016/j.pbb.2009.04.015 19409923

[B38] Nigg J. T. Wong M. M. Martel M. M. Jester J. M. Puttler L. I. Glass J. M. (2006). Poor response inhibition as a predictor of problem drinking and illicit drug use in adolescents at risk for alcoholism and other substance use disorders. *J. Am. Acad. Child Adolesc. Psychiatry* 45 468–475. 10.1097/01.chi.0000199028.76452.a9 16601652

[B39] NSDUH (2016). *2015 National Survey on Drug Use and Health: Detailed Tables*. Rockville, MD: Substance Abuse and Mental Health Services Administration. 30199192

[B40] O’Malley P. M. Johnston L. D. (2002). Epidemiology of alcohol and other drug use among American college students. *J. Stud. Alcohol Suppl.* 14 23–39. 10.15288/jsas.2002.s14.23 12022728

[B41] Patton J. M. Stanford M. S. Barratt E. S. (1995). Factor structure of the Barratt Impulsiveness Scale. *J. Clin. Psychol.* 51 768–774. 10.1002/1097-4679(199511)51:6<768::AID-JCLP2270510607>3.0.CO;2-1 8778124

[B42] Pruim R. H. Mennes M. Van Rooij D. Llera A. Buitelaar J. K. Beckmann C. F. (2015). Ica-Aroma: a robust ICA-based strategy for removing motion artifacts from fMRI data. *Neuroimage* 112 267–277. 10.1016/j.neuroimage.2015.02.064 25770991

[B43] Ridderinkhof K. R. Van Den Wildenberg W. P. Segalowitz S. J. Carter C. S. (2004). Neurocognitive mechanisms of cognitive control: the role of prefrontal cortex in action selection, response inhibition, performance monitoring, and reward-based learning. *Brain Cogn.* 56 129–140. 10.1016/j.bandc.2004.09.016 15518930

[B44] Rubia K. Smith A. B. Taylor E. Brammer M. (2007). Linear age-correlated functional development of right inferior fronto-striato-cerebellar networks during response inhibition and anterior cingulate during error-related processes. *Hum. Brain Mapp.* 28 1163–1177. 10.1002/hbm.20347 17538951 PMC6871440

[B45] Saunders J. B. Aasland O. G. Babor T. F. De La Fuente J. R. Grant M. (1993). Development of the alcohol use disorders identification Test (AUDIT): WHO collaborative project on early detection of persons with harmful alcohol consumption–II. *Addiction* 88 791–804. 10.1111/j.1360-0443.1993.tb02093.x 8329970

[B46] Schulenberg J. E. Maggs J. L. (2002). A developmental perspective on alcohol use and heavy drinking during adolescence and the transition to young adulthood. *J. Stud. Alcohol Suppl.* 14 54–70. 10.15288/jsas.2002.s14.54 12022730

[B47] Sher K. J. Martin E. D. Wood P. K. Rutledge P. C. (1997). Alcohol use disorders and neuropsychological functioning in first-year undergraduates. *Exp. Clin. Psychopharmacol.* 5 304–315. 10.1037/1064-1297.5.3.304 9260079

[B48] Silveri M. M. Cohen-Gilbert J. Crowley D. J. Rosso I. M. Jensen J. E. Sneider J. T. (2014). Altered anterior cingulate neurochemistry in emerging adult binge drinkers with a history of alcohol-induced blackouts. *Alcoholism* 38 969–979. 10.1111/acer.12346 24512596 PMC4465537

[B49] Silveri M. M. Dager A. D. Cohen-Gilbert J. E. Sneider J. T. (2016). Neurobiological signatures associated with alcohol and drug use in the human adolescent brain. *Neurosci. Biobehav. Rev.* 70 244–259. 10.1016/j.neubiorev.2016.06.042 27377691 PMC5494836

[B50] Smith E. E. Jonides J. (1999). Storage and executive processes in the frontal lobes. *Science* 283 1657–1661. 10.1126/science.283.5408.1657 10073923

[B51] Smith J. L. Mattick R. P. Jamadar S. D. Iredale J. M. (2014). Deficits in behavioural inhibition in substance abuse and addiction: a meta-analysis. *Drug Alcohol Depend.* 145 1–33. 10.1016/j.drugalcdep.2014.08.009 25195081

[B52] Smith S. M. Jenkinson M. Woolrich M. W. Beckmann C. F. Behrens T. E. Johansen-Berg H. (2004). Advances in functional and structural MR image analysis and implementation as FSL. *Neuroimage* 23 S208–S219. 10.1016/j.neuroimage.2004.07.051 15501092

[B53] Sneider J. T. Cohen-Gilbert J. E. Crowley D. J. Paul M. D. Silveri M. M. (2013). Differential effects of binge drinking on learning and memory in emerging adults. *J. Addict. Res. Therapy* S7 38–43. 10.4172/2155-6105.S7-006 PMC3881421 24404407

[B54] Sowell E. R. Thompson P. M. Tessner K. D. Toga A. W. (2001). Mapping continued brain growth and gray matter density reduction in dorsal frontal cortex: inverse relationships during postadolescent brain maturation. *J. Neurosci.* 21 8819–8829. 11698594 10.1523/JNEUROSCI.21-22-08819.2001 PMC6762261

[B55] Spear L. P. (2000). The adolescent brain and age-related behavioral manifestations. *Neurosci. Biobehav. Rev.* 24 417–463. 10.1016/S0149-7634(00)00014-2 10817843

[B56] Spinella M. (2004). Neurobehavioral correlates of impulsivity: evidence of prefrontal involvement. *Int. J. Neurosci.* 114 95–104. 10.1080/00207450490249347 14660071

[B57] Steinberg L. (2005). Cognitive and affective development in adolescence. *Trends Cogn. Sci.* 9 69–74. 10.1016/j.tics.2004.12.005 15668099

[B58] Stevens M. C. Kiehl K. A. Pearlson G. D. Calhoun V. D. (2007). Functional neural networks underlying response inhibition in adolescents and adults. *Behav. Brain Res.* 181 12–22. 10.1016/j.bbr.2007.03.023 17467816 PMC2266817

[B59] Townshend J. M. Duka T. (2005). Binge drinking, cognitive performance and mood in a population of young social drinkers. *Alcoholism* 29 317–325. 10.1097/01.ALC.0000156453.05028.F5 15770105

[B60] Van Duijvenvoorde A. C. Peters S. Braams B. R. Crone E. A. (2016). What motivates adolescents? Neural responses to rewards and their influence on adolescents’ risk taking, learning, and cognitive control. *Neurosci. Biobehav. Rev.* 70 135–147. 10.1016/j.neubiorev.2016.06.037 27353570

[B61] Van Veen V. Carter C. S. (2002). The anterior cingulate as a conflict monitor: fMRI and ERP studies. *Physiol. Behav.* 77 477–482. 10.1016/S0031-9384(02)00930-7 12526986

[B62] Verdejo-Garcia A. Bechara A. Recknor E. C. Perez-Garcia M. (2007). Negative emotion-driven impulsivity predicts substance dependence problems. *Drug Alcohol Depend.* 91 213–219. 10.1016/j.drugalcdep.2007.05.025 17629632

[B63] Weissenborn R. Duka T. (2003). Acute alcohol effects on cognitive function in social drinkers: their relationship to drinking habits. *Psychopharmacology* 165 306–312. 10.1007/s00213-002-1281-1 12439627

[B64] Wetherill R. R. Squeglia L. M. Yang T. T. Tapert S. F. (2013). A longitudinal examination of adolescent response inhibition: neural differences before and after the initiation of heavy drinking. *Psychopharmacology* 230 663–671. 10.1007/s00213-013-3198-2 23832422 PMC3840110

[B65] Whiteside S. P. Lynam D. R. (2003). Understanding the role of impulsivity and externalizing psychopathology in alcohol abuse: application of the UPPS impulsive behavior scale. *Exp. Clin. Psychopharmacol.* 11 210–217. 10.1037/1064-1297.11.3.210 12940500

[B66] Worhunsky P. D. Dager A. D. Meda S. A. Khadka S. Stevens M. C. Austad C. S. (2016). A preliminary prospective study of an escalation in ‘maximum daily drinks’, fronto-parietal circuitry and impulsivity-related domains in young adult drinkers. *Neuropsychopharmacology* 41 1637–1647. 10.1038/npp.2015.332 26514582 PMC4832027

